# SMARCA2-regulated host cell factors are required for MxA restriction of influenza A viruses

**DOI:** 10.1038/s41598-018-20458-2

**Published:** 2018-02-01

**Authors:** Dominik Dornfeld, Alexandra H. Dudek, Thibaut Vausselin, Sira C. Günther, Judd F. Hultquist, Sebastian Giese, Daria Khokhlova-Cubberley, Yap C. Chew, Lars Pache, Nevan J. Krogan, Adolfo Garcia-Sastre, Martin Schwemmle, Megan L. Shaw

**Affiliations:** 1 Institute of Virology, Medical Center, University of Freiburg, 79104 Freiburg, Germany; 2 Spemann Graduate School of Biology and Medicine, University of Freiburg, 79104 Freiburg, Germany; 3 Faculty of Medicine, University of Freiburg, 79104 Freiburg, Germany; 4 Faculty of Biology, University of Freiburg, 79104 Freiburg, Germany; 5 Department of Microbiology, Icahn School of Medicine at Mount Sinai, New York, NY 10029 USA; 6 Global Health and Emerging Pathogens Institute, Icahn School of Medicine at Mount Sinai, New York, NY 10029 USA; 7 Department of Medicine, Division of Infectious Diseases, Icahn School of Medicine at Mount Sinai, New York, NY 10029 USA; 8 Zymo Research Corp, Irvine, CA 92614 USA; 9 Quantitative Biosciences Institute, QBI, Department of Cellular and Molecular Pharmacology, University of California, San Francisco, San Francisco, CA 94158 USA; 10 J. David Gladstone Institutes, San Francisco, CA 94158 USA; 11 Sanford Burnham Prebys Medical Discovery Institute, Infectious and Inflammatory Disease Center, 10901 North Torrey Pines Road, La Jolla, CA 92037 USA

## Abstract

The human interferon (IFN)-induced MxA protein is a key antiviral host restriction factor exhibiting broad antiviral activity against many RNA viruses, including highly pathogenic avian influenza A viruses (IAV) of the H5N1 and H7N7 subtype. To date the mechanism for how MxA exerts its antiviral activity is unclear, however, additional cellular factors are believed to be essential for this activity. To identify MxA cofactors we performed a genome-wide siRNA-based screen in human airway epithelial cells (A549) constitutively expressing MxA using an H5N1 reporter virus. These data were complemented with a proteomic screen to identify MxA-interacting proteins. The combined data identified SMARCA2, the ATPase subunit of the BAF chromatin remodeling complex, as a crucial factor required for the antiviral activity of MxA against IAV. Intriguingly, our data demonstrate that although SMARCA2 is essential for expression of some IFN-stimulated genes (ISGs), and the establishment of an antiviral state, it is not required for expression of MxA, suggesting an indirect effect on MxA activity. Transcriptome analysis of SMARCA2-depleted A549-MxA cells identified a small set of SMARCA2-regulated factors required for activity of MxA, in particular IFITM2 and IGFBP3. These findings reveal that several virus-inducible factors work in concert to enable MxA restriction of IAV.

## Introduction

Influenza A viruses (IAV) are severe human pathogens all originating from their avian reservoir. Human-adapted IAV are the cause of annual epidemics but rarely also cause pandemics with millions of people succumbing to infection^1^. The recent emergence of “bird flu” viruses of the H5N1 and H7N9 subtype, with fatality rates among humans ranging up to ~50%, raises concern that these avian viruses might acquire the ability to transmit from human-to-human and initiate the next pandemic^1–3^.

Influenza A viruses have a segmented RNA genome of negative polarity with each genomic segment organized as a viral ribonucleoprotein (vRNP) consisting of the viral RNA, the three polymerase subunits, PB2, PB1 and PA, and the viral nucleoprotein (NP) which is required for encapsidation of the viral RNA^4^. Following virus entry, the viral NP mediates rapid nuclear translocation of vRNPs, a property enabling IAV to evade cytosolic pattern recognition receptors and granting access to the nuclear splicing machinery required for the expression of certain viral genes^4,5^. Initially, the IAV polymerase synthesizes mRNA transcripts which are translated into viral proteins at cytosolic ribosomes. Once a sufficient amount of newly synthesized viral protein (in particular PB2, PB1, PA and NP) has been made and imported into the nucleus, the viral polymerase switches to synthesis of complementary RNA which is encapsidated into complementary RNPs (cRNPs) during this process. These cRNPs in turn serve as intermediates for synthesis of new vRNPs which are finally exported from the nucleus to be packaged into new viral particles at the plasma membrane^4^.

An intact innate immune response is critical for restriction of viral replication and survival, as exemplified not only in animal experiments but also by more severe disease progression seen in young children with mutations in crucial innate immune genes^6,7^. After recognition of pathogen-associated molecular patterns, such as viral RNA, the cell responds by secreting type I and III interferons. These interferons bind to their respective receptors leading to JAK-STAT signaling and eventually to induction of several hundred interferon-stimulated genes (ISGs), of which some have direct effector activity against particular viruses^7^. The speed of the antiviral response is critical to prevent further virus spread. Therefore, most ISG promoters, which would otherwise be covered by nucleosomes and not be available for immediate access, are “primed” for rapid transcriptional activation through the bound SWI/SNF chromatin remodeling complex (BAF complex)^8^. The promotor-bound BAF complex not only facilitates rapid induction but also basal level expression and, upon stimulation, induction of ISGs to a higher extent.

Among many other ISGs, human MxA has been shown to be particularly potent in inhibiting IAV replication *in vitro* as well as *in vivo*^9–11^. Its importance is further emphasized by the fact that all human-adapted IAVs, including all pandemic viruses, have evolved partial resistance towards MxA antiviral activity through mutations in their NP^9^. MxA belongs to the family of large dynamin-like GTPases and localizes to the cytoplasm, in contrast to IAV replication which occurs in the nucleus. To date, the mechanism by which MxA inhibits IAV replication is still unclear and somewhat controversial^12,13^. However, there is general agreement that a very early step of the IAV replication cycle is affected^14–16^.

Avian influenza viruses, including the subtypes H5N1 and H7N7, are highly sensitive to MxA, but the finding that human-adapted IAV are able to partially escape the antiviral effect of MxA through mutations in NP suggested that restriction might be mediated through direct physical binding of IAV NP to MxA^9–11,17,18^. However, while NP of the closely related Thogoto virus (THOV) can be co-precipitated with MxA, similar approaches with IAV NP have failed to show a convincing interaction^19^. Therefore, it is hypothesized that the IAV NP-MxA interaction is of low affinity and may require the presence of additional host cell factors^13,19,20^.

THOV replication was shown to be inhibited by MxA through a block in nuclear translocation of incoming vRNPs^21^ and Xiao and colleagues recently demonstrated that a similar mode of action might apply to IAV vRNPs^16^. However, their experimental setup required IFN pretreatment in addition to MxA overexpression, suggesting that additional ISGs are required for MxA restriction of influenza A viruses. In line with that, the related human paralog of MxA, MxB, similarly depends on host cell factors for its anti-HIV-1 activity, as it loses its ability to prevent viral DNA from entering the nucleus and integrating into the genome in the absence of cyclophilin A^22^.

Here, we conducted a genome-wide siRNA screen and a proteomic screen to identify potential MxA cofactors required for the antiviral activity of human MxA, and we identified SMARCA2 as essential for the antiviral effect of MxA against H5N1 and H7N7 viruses. SMARCA2 is the ATPase subunit of the SWI/SNF chromatin remodeling complex (BAF complex), the complex known to reside on promoters of many ISGs thereby facilitating their induction. Our data show that SMARCA2 is required for the induction of an antiviral state in IFN-treated cells but that induction of MxA *per se* is not affected by the absence of SMARCA2. A transcriptome analysis of SMARCA2-depleted A549-MxA cells identified a large number of SMARCA2-regulated genes, of which many are ISGs. Several of these factors, in particular IFITM2 and IGFBP3, were required for efficient inhibition of viral replication in A549-MxA cells. Therefore, our data demonstrate that several SMARCA2-dependent ISGs act in concert to facilitate the antiviral activity of MxA against influenza A viruses.

## Results

### Identification of host cell factors required for the antiviral activity of MxA

To identify factors required for the antiviral activity of MxA, we developed a genome-wide siRNA screening assay on a well-established A549 human lung epithelial cell line stably over-expressing human MxA (A549-MxA)^16^. To easily monitor virus replication a reporter virus was generated based on the highly MxA-sensitive influenza A/Vietnam/1203/2004 (H5N1) virus^17^ by incorporating a Renilla (RL) luciferase reporter construct into the NS segment (H5N1-RL) as previously described by Reuther and colleagues (Supplementary Figure S1a)^23^. Furthermore, to allow utilization of this highly pathogenic avian influenza (HPAI) virus under biosafety level 2 (BSL-2) conditions several positively charged amino acids were removed from the hemagglutinin (HA) cleavage site to render it monobasic, and thus classifying the virus as a low pathogenic avian virus (Supplementary Figure S1b)^24,25^. In the presence of MxA, virus replication is inhibited resulting in low levels of RL activity, but upon transfection of an siRNA targeting MxA (silencing the MxA-encoding gene MX1), virus replication is restored and an increase in RL activity is observed (Supplementary Figure S2a). The goal of the siRNA screen was to identify siRNAs that restore virus replication in the presence of MxA, so siMX1 served as the positive control and a non-targeting (NT) siRNA as the negative control. The assay was miniaturized to 384-well format and the quality was assessed by measuring the strictly standardized mean difference (SSMD)^26^, which accounts for the assay window and variability. The SSMD was calculated as 9.4, confirming a high quality assay suitable for high-throughput screening (Supplementary Figure S2a).

**Figure 1:**
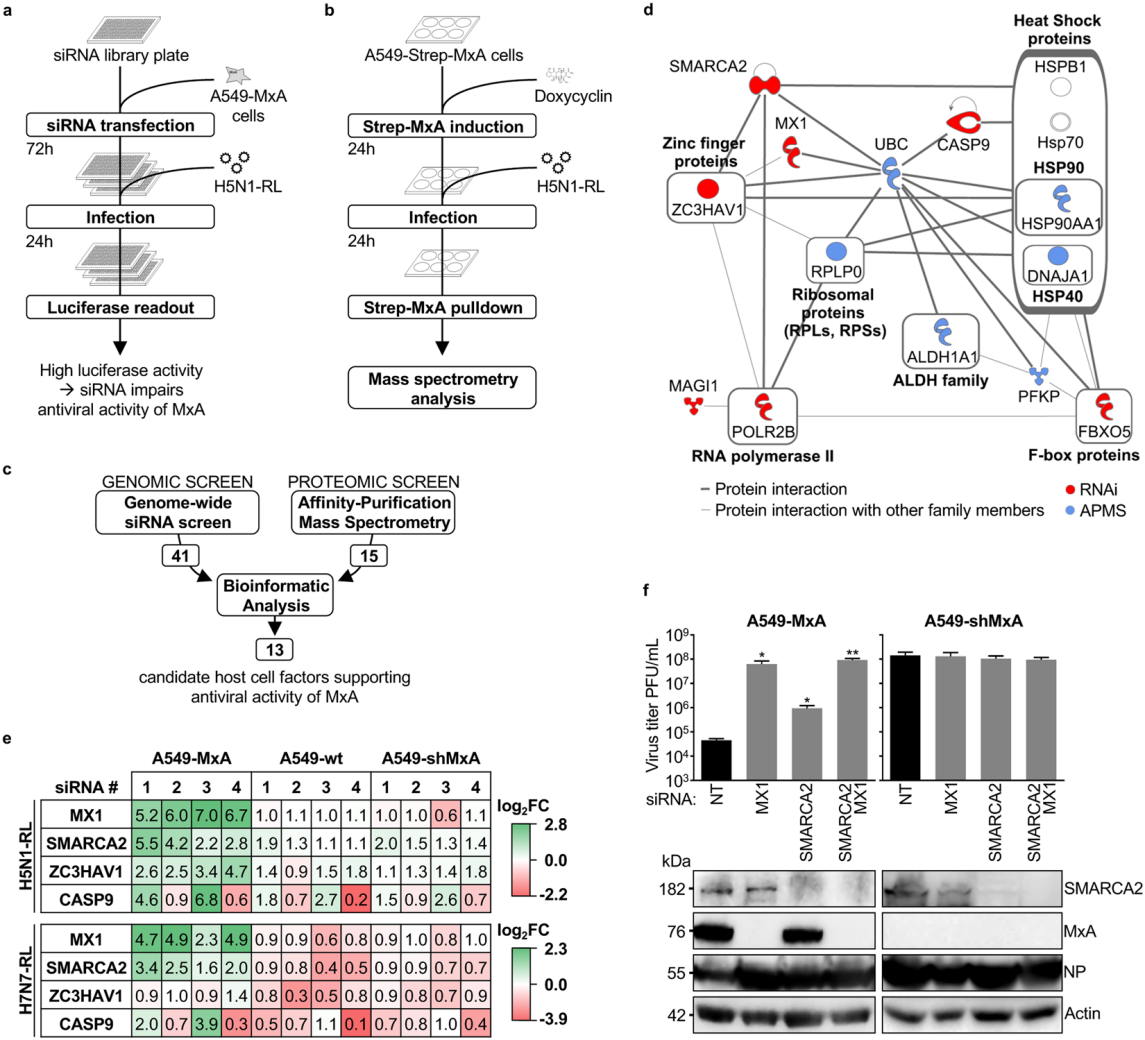
RNAi and proteomic screens identify SMARCA2 as potential MxA co-factor. (**a**) For the RNAi screen A549-MxA cells were transfected in triplicate with siRNA library pools in 384-well plates for 72 hours. Cells were then infected with H5N1-RL virus (MOI of 8) and luciferase activity determined 24 h later. (**b**) For the proteomic screen A549-Strep-MxA and A549-Strep-GFP cells were treated with 1 μg/mL of doxycycline for 24 hours to induce the expression of streptavidin-tagged MxA (Strep-MxA) or GFP (Strep-GFP). Then, cells were infected with H5N1-RL (MOI of 8) for 24 hours and Strep-tagged proteins were affinity purified and analyzed by mass spectrometry. (**c**) Results from (a) and (b) were analyzed using Ingenuity® Pathway Analysis (IPA®, Qiagen) and a core set of 13 enriched factors was identified. (**d**) The protein-protein interaction network of the 13 factors identified through bioinformatic analysis. (**e**) The effects of SMARCA2, ZC3HAV1 and CASP9 depletion on virus replication was assessed in A549-MxA cells, A549 wild type cells (A549-wt) or A549-shMxA cells. Cells were transfected with a non-targeting control siRNA (NT) or with one of four siRNAs targeting MxA (MX1), CASP9, SMARCA2 or ZC3HAV1. 72 h post transfection cells were infected with H5N1-RL or H7N7-RL (MOI of 0.3) and 24 h later virus reporter activity was measured. The heatmap indicates increased (green) or decreased (red) viral replication as compared to the NT control siRNA (white) and demonstrates the data of 3 independent experiments (4 technical replicates per experiment). (**f**) siRNA depletion in A549-MxA cells was performed as described above for MX1, SMARCA2 (the most efficient siRNA: GE Dharmacon D-017253-01) or a combination of MX1 and SMARCA2. 72 h post transfection cells were infected with A/Thailand/1(KAN-1)/2004 (H5N1) (MOI of 0.001). At 36 h post infection virus titers were determined by plaque assay. Error bars indicate the standard error of the mean of three independent experiments. Student’s t-test was performed to determine the *P* value. *P < 0.05, **P < 0.01. Expression of SMARCA2, MxA, NP and Actin was determined by western blot. Full-length blots are presented in Supplementary Figure S8a and S8b. Log2FC = Log2 Fold Change.

An arrayed genome-wide siRNA library of siRNA pools (4 siRNAs per gene) targeting a total of 18119 human genes was transfected into A549-MxA cells in triplicate. 72 hours post transfection cells were infected with the H5N1-RL reporter virus at a multiplicity of infection (MOI) of 8, and 24 h later luciferase activity was determined utilizing Renilla-Glo (Promega) substrate (Fig. 1a). The SSMD was used to validate the quality of each plate (SSMD > 3)^26^ and hits were selected based on an average Z-score >2 across triplicates (Supplementary Figure S2b,c). This resulted in the identification of 276 primary hits (Supplementary Table S1). To exclude off-target effects those hits were further validated in a secondary siRNA screen by individually transfecting the 4 siRNAs from each pool (Supplementary Figure S2b). The SSMD was used to validate each plate and the results were analyzed using Redundant siRNA Activity (RSA) statistical analysis^27^ which assigns a p-value to each gene based on the effect of its four siRNAs. Hits were selected based on a p-value < 0.05. Using these methods, we identified 41 genes, including the MxA-encoding gene MX1, whose knockdown led to a significant (p-value < 0.05) increase in virus growth in A549-MxA cells (Supplementary Table S2).

### Identification of host cell factors physically associated with MxA

In parallel with the RNAi-mediated approach of identifying host factors required for MxA activity we employed a second strategy aimed at identifying host cell factors that interact with MxA. Experimental conditions were largely maintained to allow a combined bioinformatic analysis of siRNA screening and proteomic screening data at a later step. The antiviral activity of Mx proteins is not affected by the addition of amino-terminal tags^28,29^. Therefore A549 cells expressing amino-terminally streptavidin-tagged MxA (Strep-MxA) under control of a doxycycline-inducible promoter were generated alongside control cells expressing Strep-GFP. Strep-MxA and Strep-GFP were induced with doxycycline for 24 hours and the cells were then either infected with H5N1-RL or mock infected in triplicate. 24 hours post infection, cells were lysed and the Strep-tagged proteins precipitated by affinity purification with Strep-Tactin sepharose beads. Precipitated proteins were digested and identified by mass (Fig. 1b, Supplementary Figure S3). Mass spectrometry files were searched using MAXQuant and statistical analysis performed using MSStats^30,31^. None of the factors were virus-encoded. 15 host cell factors qualified as high-confidence hits based on enrichment with the MxA bait vs. the GFP bait during H5N1-RL infection (Log2FC > 2; p-value < 0.05 under statistical conditions to minimize false negatives or Log2FC > 2; p-value < 0.1 under statistical conditions to minimize false positives) (Supplementary Table S3).

### Bioinformatic analysis of the combined RNAi and proteomic datasets

In the genomic and proteomic screens, we identified 41 and 15 host cell factors, respectively. Although comparison of the resulting hits shows no direct overlap, using Ingenuity Pathway Analysis (IPA®, Qiagen) we were able to identify a network of 13 host cell factors (Supplementary Table S4), including MxA, that are significantly enriched amongst the dataset when analyzed for representation in canonical pathways, upstream regulators and protein-protein interaction networks (p-value < 0.05, Fig. 1c,d, Supplementary Figure S4a). Within this network of 13 factors, 7 (including MxA) were identified in the siRNA screen and 6 in the proteomic screen. To validate the requirement of these 13 factors for MxA antiviral activity, we transfected A549-MxA cells with siRNAs targeting the respective gene (4 siRNAs per gene). Following infection with H5N1-RL, luciferase activity was measured to assess the antiviral effect. We considered a factor to be validated if at least 2 out of 4 siRNAs significantly increased virus replication by 90% (Log2FC ≥ 0.93, p-value < 0.05) compared to a non-targeting (NT) control siRNA (Supplementary Figure S4b). Those criteria were only met by SMARCA2, ZC3HAV1 (also known as ZAP) and CASP9. As these data were generated in the presence of MxA they suggest that SMARCA2, ZC3HAV1 and CASP9 are required for efficient MxA restriction of H5N1, however, they may also have an antiviral effect independent of MxA.

### SMARCA2 and CASP9 have an antiviral role only in the presence of MxA

ZC3HAV1 is a restriction factor with a broad antiviral spectrum. However, its activity against IAV is only modest^32,33^ and there is no information about its relevance to MxA restriction. Similarly, for CASP9, an initiator caspase required for the apoptotic pathway, neither association with MxA nor IAV has been shown. SMARCA2 is an ATPase subunit of the BAF chromatin remodeling complex, a complex required for the induction of many genes, including interferon-stimulated genes (ISGs) with anti-IAV activity^34^. To verify that SMARCA2, ZC3HAV1 and CASP9 are required for the antiviral activity of MxA, but do not act as MxA-independent restriction factors, we made use of A549 wild type cells (A549-wt) and the established cell line A549-shMxA which stably overexpresses a short hairpin RNA targeting MxA (shMxA)^16^. Due to the stable expression of shMxA even MxA induced by viral infection is expected to be immediately silenced. Upon infection with H5N1-RL or a Renilla-expressing reporter virus based on the MxA-sensitive IAV strain A/seal/Massachusetts/1/1980^10,11^ (H7N7-RL), high reporter activity was observed in A549-wt and A549-shMxA cells but replication was considerably reduced in A549-MxA cells due to the presence of MxA (Supplementary Figure S5a). siRNA knockdown of MxA (MX1), CASP9 and SMARCA2 increased reporter activity of both viruses by up to ~6-fold and at least 2-fold in A549-MxA cells (2 out of 4 siRNAs increasing reporter activity) (Fig. 1e and Supplementary Figure S5b). On the other hand, in A549-shMxA and A549-wt cells such an increase was not observed upon RNAi-mediated depletion. These data suggest that MxA requires the presence of SMARCA2 and CASP9 to efficiently restrict influenza A viruses and in the absence of MxA, SMARCA2 and CASP9 do not display antiviral properties.

### Silencing of SMARCA2 increases growth of H5N1 virus in A549-MxA cells

To provide evidence that SMARCA2 is required for the antiviral activity of MxA not only in the context of a reporter virus but also with wild type virus infection, we performed siRNA knockdown as before and infected A549-MxA cells and A549-shMxA cells with the MxA-sensitive H5N1 strain A/Thailand/1(KAN-1)/2004 at an MOI of 0.001^9,18^. 36 hours post infection viral titers were measured by plaque assay and knockdown efficiency was determined by western blot analysis (Fig. 1f). Silencing of MxA (MX1) in A549-MxA cells elevated the virus titer by approximately 3 log_10_ compared to cells treated with the non-targeting control siRNA (NT), in which the virus was restricted to ~5 × 10^4^ PFU/mL due to MxA overexpression. Knockdown of SMARCA2 using the most efficient siRNA (Supplementary Figure S5b,d) resulted in >1 log_10_ increase in viral titer relative to siNT in A549-MxA cells (Fig. 1f). The combination of SMARCA2 and MxA knockdown did not result in a further increase of viral titer over the level of siMxA alone, which is similar to that observed with both wild-type and reporter viruses in siSMARCA2-treated A549-shMxA cells (Fig. 1e,f, Supplementary Figure S5b). In summary these data confirm that SMARCA2 is required for efficient antiviral activity of MxA in the context of an H5N1 wild type virus infection.

### SMARCA2 is required for ISG induction but not for expression of MxA

**Figure 2:**
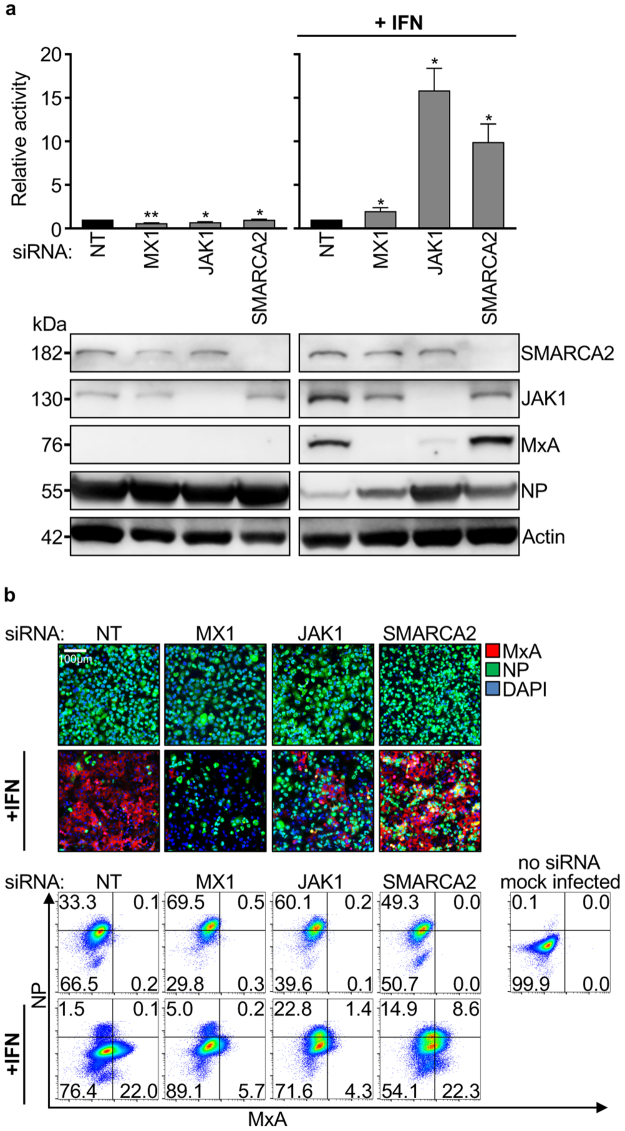
SMARCA2 is required for ISG induction but not for expression of MxA. (**a**) A549-wt cells were transfected with a non-targeting (NT) siRNA, or siRNAs targeting MxA (MX1), JAK1, or SMARCA2 (GE Dharmacon D-017253-01). 48 h post transfection the cells were either treated, or not, with IFN-α (1000 U/mL) and 24 h later were infected with H7N7-RL at an MOI of 0.3. Luciferase activity was measured 24 h post infection to determine virus reporter activity or cells were lysed and subjected to western blot analysis of the indicated proteins. Full-length blots are presented in Supplementary Figure S8c. Virus reporter activity was determined using 6 technical replicates. All data were normalized to the respective NT control. Error bars indicate the standard error of the mean of three independent experiments. Student’s t-test was performed to determine the *P* value. *P < 0.05, **P < 0.01. (**b**) A549-wt cells were treated as described in (a) but infected at an MOI of 1 for 24 hours. For immunofluorescence analysis (upper panel) MxA (red) and NP (green) were stained with specific antibodies. DAPI was used to counterstain the nucleus (blue). Lower panel: The number of cells positive for NP and MxA was quantified using flow cytometry analysis.

SMARCA2 is the ATPase subunit of the BAF chromatin remodeling complex. This complex has been shown to have chromatin remodeling activity either in conjunction with SMARCA2 or another ATPase subunit, SMARCA4, depending on the development stage and cell type investigated^35–37^. In HeLa cells the SMARCA4-containing BAF complex was shown to be required for induction of 90% of type-I interferon-stimulated genes (ISGs)^8^. However, A549 cells do not express SMARCA4 protein (Supplementary Figure S6a, ref.^38^), so we aimed to investigate whether SMARCA2 is involved in ISG expression in these cells. A549-wt cells were transfected with siRNAs targeting MxA, JAK1, or SMARCA2 and then treated, or not, with 1000 U/mL IFN-α 48 h after siRNA transfection. 24 h after IFN treatment, the cells were challenged with H7N7-RL. In the absence of IFN no enhancement of viral replication was observed relative to the non-targeting (NT) control siRNA (Fig. 2a). In IFN-treated A549-wt cells, however, silencing of JAK1, which is a major signal transducer of IFN signaling^39,40^ and required for the IFN-mediated induction of all ISGs, led to a significant increase in viral replication (~15-fold), while knockdown of the single ISG, MxA, elevated viral replication by about 2-fold (Fig. 2a). Intriguingly, knockdown of SMARCA2 resulted in a ~10-fold increase in virus replication, suggesting that SMARCA2 is indeed required for the establishment of an IFN-inducible antiviral state in A549 cells.

One explanation may be that, as for JAK1, SMARCA2 is required for MxA expression along with other ISGs. Indeed, in the case of JAK1 knockdown, MxA expression is substantially diminished as determined by western blot analysis (Fig. 2a). But surprisingly SMARCA2 depletion does not alter MxA induction by IFN. This finding leads us to conclude that SMARCA2 is required for robust induction of ISG(s) other than MxA which in turn support the antiviral activity of MxA. Thus, in the absence of SMARCA2, cells expressing MxA should show increased susceptibility to IAV infection. Analysis of SMARCA2-silenced A549-wt cells pretreated with 1000 U/mL IFN-α 24 h prior to H7N7-RL infection revealed that in fact 8.6% of cells were positive for both MxA and viral nucleoprotein (NP), compared to 0.1% in the non-targeting (NT) control knockdown (Fig. 2b and Supplementary Figure S6b). Based on these findings we hypothesize that SMARCA2 facilitates the induction of one or several ISG(s) which in turn support the antiviral activity of MxA against IAV.

### Identification of SMARCA2-regulated factors supporting MxA activity

**Figure 3:**
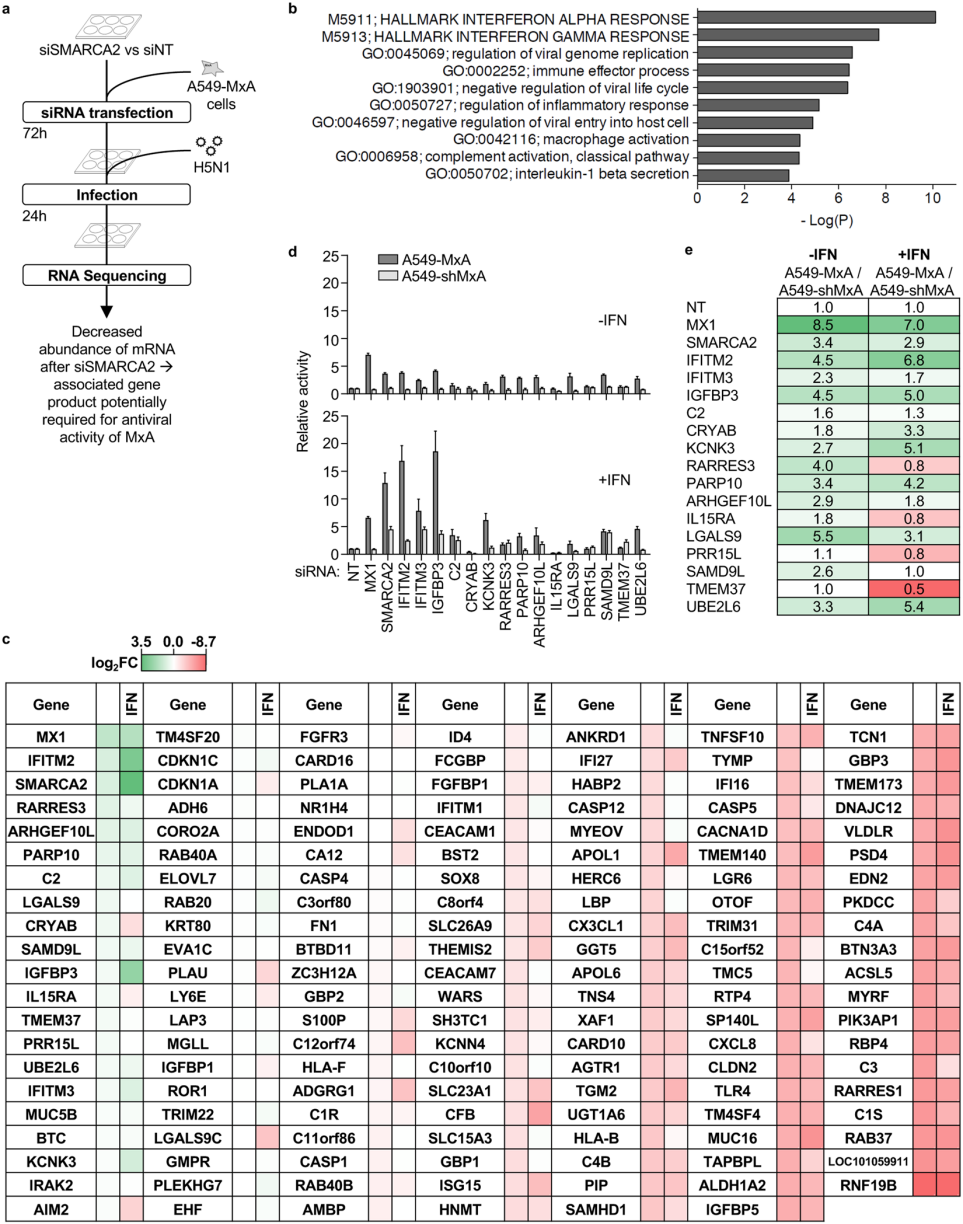
Identification of SMARCA2-regulated MxA cofactors. (**a**) A549-MxA were transfected with an siRNA targeting SMARCA2 (Dharmacon D-017253-01) or a non-targeting control siRNA (siNT). 72 h post siRNA transfection cells were infected with H5N1 strain A/Thailand/1(KAN-1)/2004 at an MOI of 1 and harvested using DNA/RNA shield (Zymo Research). RNA extraction, rRNA depletion and analysis of the respective transcriptome was performed by Zymo Research as part of the EpiQuest TM service. (**b**) Gene ontology term enrichment analysis of host mRNAs differentially regulated by depletion of SMARCA2 in infected A549-MxA cells. (**c**) Host cell factors whose mRNA abundance was strongly decreased after SMARCA2 knockdown were silenced in A549-MxA cells using siRNA pools. 66 h after siRNA transfection A549-MxA cells were either treated, or not, with IFN-α (25 U/mL) and infected 6 h later with H7N7-RL reporter virus (MOI = 0.3). After 24 h virus reporter activity was measured using Renilla-Glo (Promega) substrate (3 technical replicates). The heatmap indicates increased (green) or decreased (red) viral replication as compared to the non-targeting siRNA control (white). (**d**) Knockdown and infection was performed as in (**c**) but experiments were extended to A549-shMxA cells and only siRNAs resulting in strong increase of viral reporter activity were used for knockdown (see (**c**)) (6 technical replicates). (**e**) To assess MxA-dependent effects, the ratios of reporter virus activities (from (**d**)) between A549-MxA cells and A549-shMxA cells were calculated and are presented as a heat map (High ratios in green and low ratios in red). Log2FC = Log2 Fold Change.

To identify SMARCA2-regulated ISGs we performed a transcriptome analysis of SMARCA2-depleted A549-MxA cells. Either siSMARCA2- or siNT-treated A549-MxA cells were infected with wild type H5N1 virus (A/Thailand/1(KAN-1)/2004) at an MOI of 1 (Fig. 3a). 24 h post infection cells were harvested, RNA was extracted and after rRNA depletion subjected to mRNA sequencing. 140 host mRNAs were identified as being downregulated by at least 10-fold and as much as 126-fold in siSMARCA2-treated A549-MxA cells (Supplementary Table S5). Gene ontology (GO) term enrichment analysis revealed that the dataset was highly enriched in gene categories associated with IFN alpha and gamma responses (Fig. 3b) which is in line with our previous finding that SMARCA2 is required for establishment of an antiviral state (Fig. 2).

A total of 144 genes were selected for validation based on the extent of their downregulation after SMARCA2 depletion (top 104 genes with Log_2_FC < −3.57, corresponding to >12-fold downregulation) and their identification as ISGs (interferome.org) in A549 cells (50 genes with Log_2_FC < −2) as well as other cell lines (48 genes with Log_2_FC < −3.5). Another 15 handpicked genes were added, including controls such as SMARCA2, and finally, pseudogenes were filtered out. To investigate which of the SMARCA2-regulated factors are required for the antiviral activity of MxA, siRNA pools were used to silence each gene in A549-MxA cells which were subsequently infected with H7N7-RL. To ensure sufficient induction of the selected factors (and thus allow for knockdown) the cells received 25 U/mL IFN-α 6 h prior to infection, which was the minimal amount found to reduce viral reporter activity (indicative of ISG induction) without diminishing it completely (Supplementary Figure S7a). None of the siRNAs had cytotoxic effects (Supplementary Figure S7b). The effect of each siRNA pool on virus replication +/− IFN was determined and ranked according to the strongest increase in viral replication (green) in the absence of IFN (Fig. 3c, Supplementary Figure S7b). As expected, although the general pattern was similar, greater effects were observed in the presence of IFN, with MxA depletion enhancing replication by 5.9-fold and knockdown of SMARCA2 by 11.2-fold. Several factors displayed clear effects, such as IGFBP3, which increased viral replication by 8-fold upon silencing. Most prominently however, knockdown of the ISG IFITM2 resulted in a 9.5-fold increase in viral replication. Knockdown of IFITM3, on the other hand, which is a relative of IFITM2 and known to be a potent IAV restriction factor^41^, showed only a 3.3-fold increase in virus replication under MxA overexpression conditions. In the absence of MxA (A549-shMxA cells) the antiviral effect of IFITM3 is more visible, and stronger than that of IFITM2, when cells are pre-stimulated with IFN (Fig. 3d). Generally, the replication-enhancing effects upon knockdown of the most potent SMARCA2-regulated factors appear to depend on the presence of MxA for full effect, as no, or only minor, increases in H7N7-RL reporter activity were observed in A549-shMxA cells, e.g. 2.5-fold with IFITM2 and 3.7-fold with IGFBP3 (Fig. 3d). To more easily assess whether factors depend on MxA we calculated the ratio of the relative reporter activity between A549-MxA and A549-shMxA cells either with or without IFN-pretreatment and considered all factors with a ratio >2 (Fig. 3e). The factors with the strongest evidence for being MxA co-factors are: ARHGEF10L, IFITM2, IFITM3, IGFBP3, KCNK3, PARP10, RARRES3, SAMD9L and UBE2L6. In summary these data identify several factors, in particular IFITM2 and IGFBP3, as SMARCA2-regulated, virus-inducible factors required for IAV restriction by MxA.

## Discussion

The MxA GTPase is a potent IFN-inducible restriction factor, inhibiting early steps of the influenza A virus (IAV) replication cycle. In the absence of MxA the ribonucleoprotein-organized viral genome is rapidly transported to the nucleus where viral transcription and genome replication takes place, but this is blocked in the presence of MxA. Although postulated^16^, it remains to be shown whether other cellular factors are involved in mediating the antiviral effects of MxA. We therefore conducted parallel RNAi and protein interaction screens to identify potential MxA cofactors and identified a network of 13 factors supported by both screens. We focused on one particular factor, SMARCA2, which was validated as being required for efficient MxA restriction of both H5N1 and H7N7 IAVs.

SMARCA2 (also named BRM) is one of two mutually exclusive ATPase subunits of the evolutionary highly conserved BAF chromatin remodeling complex (also known as the SWI/SNF complex), which has been shown to be involved in regulation of gene expression, cell cycle control and tumorigenesis^35,42–50^. Interestingly, the human lung adenocarcinoma cell line, A549, used in our study does not express the other ATPase subunit, SMARCA4 (or BRG1)^38^. Loss or mutation of SMARCA4 seems to be a relatively frequent event during tumorigenesis^51,52^ and SMARCA2 can at least partially compensate for the loss of SMARCA4^53–55^. Suffice to say that by using A549 cells we could study the role of SMARCA2 specifically. We were unable to generate A549 SMARCA2 CRISPR knockout cells as cell proliferation was inhibited, which agrees with previous reports^55–57^. However, with transient knockdown we found that SMARCA2 depletion decreased the ability of IFN-treated A549 cells to establish an antiviral state. This is in agreement with previous findings that the BAF complex is required for induction of a subset of IFN-α-inducible genes^8,58,59^, although in these studies SMARCA4/BRG1 was the focus. Surprisingly, however, MxA expression is unaffected by SMARCA2 depletion, suggesting that reduced MxA antiviral function is due to the absence of one or more SMARCA2-regulated factors. Flow cytometry experiments support this by demonstrating that SMARCA2 depletion leads to an elevated number of cells positive for both viral antigen (NP) and MxA in IFN-pretreated A549 cells. These results also provide strong support to the findings of Xiao and colleagues that additional ISGs are required for full MxA activity^16^ and further show that these ISGs are probably regulated by SMARCA2. Alternatively MxA may interact with SMARCA2, although this is unlikely as neither SMARCA2 nor any other BAF component was identified as an MxA-interacting candidate in our mass spectrometry analysis. Also, SMARCA2 is a nuclear protein unlike MxA^60^.

A transcriptome analysis on IAV-infected SMARCA2-depleted A549-MxA cells identified 140 genes that strongly depend on the presence of SMARCA2. By far the most significantly enriched gene category was interferon-α response-associated genes, further validating the importance of SMARCA2 to ISG expression, even in the absence of SMARCA4. Cui and colleagues demonstrated that SMARCA4 in HeLa cells constitutively binds to the promoters of the ISGs IFITM1, IFITM2 and IFITM3 resulting in basal level expression and rapid and strong induction upon IFN-α stimulation^8^. We show that the same genes are regulated by SMARCA2 in A549 cells. Finally, we determined that 9 of these SMARCA2-regulated factors are required for full antiviral activity of MxA: ARHGEF10L, IFITM2, IFITM3, IGFBP3, KCNK3, PARP10, RARRES3, SAMD9L and UBE2L6. The application of low levels of IFN-α in these experiments allowed for a larger assay window when detecting ISGs, and this may account for why we failed to detect these factors in the primary RNAi screen. Intriguingly 6 of the 9 host cell factors are ISGs^61^ and 2 factors, KCNK3 and IGFBP3, are induced in response to IFN in virus infected cells, so may be either IFN- or virus-inducible. As expected some factors display antiviral activity in the absence of MxA too (e.g. IFITM3, IGFBP3, IFITM2) but others seem to strictly depend on the presence of MxA, such as KCNK3, PARP10 and UBE2L6. Nonetheless, in all cases, the IAV restriction potency of these factors is increased in A549-MxA cells.

The antiviral activity of MxA appears to rely particularly on the presence of IGFBP3 and IFITM2. IGFBP3 (insulin-like growth factor binding protein 3) binds insulin-like growth factors IGF-1 and IGF-2 circulating in the plasma and modulates their growth-promoting effects^62^. Furthermore, IGFBP3 can interact with the IGFBP-3R receptor thereby acting as tumor suppressor^63^ and it can be internalized resulting in alterations of cell signaling^64–67^. It is unclear how IGFBP3 could increase MxA activity and further studies will be required to determine whether it acts as an ISG, as some of our data suggest. IFITM2 belongs to a protein family, the interferon-inducible transmembrane protein family, which is known for its importance in IAV restriction. IFITM proteins are type-I and type-II IFN-inducible cell-intrinsic restriction factors and were first described to have antiviral functions in 1996 by Alber *et al*.^68^. More recently their significance in IAV restriction was discovered in a genome-wide siRNA screen^41^. However, IFITMs are also active against many other enveloped RNA viruses^41,69–77^. In humans three IFITMs are IFN-inducible and display antiviral properties, namely IFITM1, 2 and 3^78^. IAV restriction is mainly mediated by IFITM3, which has similar potency to MxA^16^, while IFITM2 shows an intermediate phenotype and IFITM1 only demonstrates minor effects^79^. For CCR5-tropic HIV-1 and HCV restriction, however, IFITM1 plays the dominant role which is at least in part due to its localization to the plasma membrane^69,71^. In the case of CCR5-tropic HIV-1 it is believed that IFITM-1 counteracts viral fusion at the plasma membrane to restrict access to the cytosol^69^. Similarly IFITM3 and IFITM2, which only differ by 12 amino acids, are thought to block fusion of IAV with the endosomal membrane^79^. Therefore the main mechanism of restriction by IFITMs is by preventing fusion of viral membranes with cellular lipid bilayers, although other mechanisms have also been proposed^80^. Here, we demonstrate that in the presence of MxA, IFITM2 is the major IFITM member required for IAV restriction. Interestingly, both IFITM2 and MxA are thought to restrict IAV at similar stages of the virus life cycle, with IFITM2 described to act in the late endosome and MxA directly after release of vRNPs from the late endosome^15,16,79^. Furthermore, MxA has been shown to associate with membranes^81,82^, therefore it is conceivable that IFITM2 and MxA act in a coordinated sequential fashion in a similar manner as was recently proposed by Narayana *et al*. for IFITM1, 2 and 3^71^. This study proposed that IFITMs would trap the virus in the endocytic pathway and eventually redirect it to lysosomal degradation. We propose that MxA might cooperate to a small extent with IFITM3 at the early endosome and more strongly with IFITM2 at the late endosome shortly before vRNP release. Intriguingly, Narayana and colleagues already hypothesized that IFITM2 may associate with unknown host factors to restrict viral replication^71^. Apart from IFITM2 and IGFBP3, several other factors were also found to show stronger antiviral effects in the presence of MxA. It is therefore possible that to achieve full viral inhibition all, or several, of the identified factors may have to act in concert with each other, as well as with MxA. Further studies will be required to investigate the individual contribution of these factors and whether a certain combination is needed to fully support the antiviral activity of MxA.

In summary this study not only identifies IFITM2, IGFBP3 and some other IFN- or virus-inducible host factors as being required for the full antiviral activity of MxA, but also further emphasizes the critical role of chromatin remodeling complexes in antiviral immune responses.

## Methods

### Cell culture

Wild type A549 human cells from lung carcinoma (ATCC® CCL-185™), A549 cells stably overexpressing MxA (A549-MxA), A549 cells stably expressing a short hairpin RNA targeting MxA (A549-shMxA)^16^ (kindly provided by Richard E. Randall, University of St Andrews, St Andrews, UK), and A549 cells stably expressing streptavidin-tagged MxA (A549-Strep-MxA) or streptavidin-tagged GFP (A549-Strep-GFP) were generated as described below and cultured at 37 °C, 5% CO2 in DMEM medium with FBS (10%) and penicillin-streptomycin (1%). Canine MDCKII cells used for virus titration were cultured under the same conditions.

### Viruses

The recombinant H5N1 virus (A/Thailand/1(KAN-1)/2004) was generated as described previously^9^. The H7N7 (A/seal/Massachusetts/1/1980) Renilla luciferase reporter virus harboring a multibasic HA cleavage site was obtained as published by Reuther and colleagues^23^. Briefly, the splice donor and splice acceptor sites in the NS gene-encoding sequence were silenced. The overlapping NS1 and the NEP open reading frames were separated and the Renilla luciferase-encoding sequence was introduced in between. To obtain 3 separated gene products porcine teschovirus-1 2A peptide-encoding sequences were introduced in between the 3 ORFs (for a more detailed description also see Fig. S1a). The same procedure was applied to generate the H5N1 (A/Vietnam/1203/2004) Renilla luciferase virus. However, in this case a virus encoding a monobasic HA cleavage site was generated by altering the cleavage site as described previously^24,25^. All recombinant viruses were plaque purified on MDCKII cells. Virus stocks were either prepared on MDCKII cells or in 8 days old embryonated chicken eggs and titers were determined by plaque assay.

### siRNA screen

A primary genome-wide screen assessing the role of 18119 genes was performed using siRNA pools of 4 siRNAs transfected into A549-MxA. A non-targeting siRNA (GE Dharmacon D-001810-10) and an MxA-targeting siRNA (Qiagen SI05459538) served as negative and positive control, respectively. The cells were seeded in 384-well plates (2800 cells/well) and reverse transfected with siRNAs (final concentration 10 nM) using DharmaFECT4 transfection reagent (0.125 µL/well). Each 384-well plate was run in triplicate. After 72 hours, the cells were infected with the H5N1 Renilla luciferase reporter virus at an MOI of 8 for 24 hours. Luciferase activity was analyzed by adding Renilla luciferase substrate (5 µl, Renilla-Glo Luciferase Assay System, Promega) and read using the Envision Multilabel plate reader (PerkinElmer). A secondary screen was performed on 276 genes by individually transfecting the 4 siRNAs from the pool used in the primary screen. The rest of the procedure was performed as described above. The siRNAs library plates were provided by GE Healthcare Dharmacon Inc. (human siGENOME SMARTpool library G-005005) and siRNAs were resuspended at a concentration of 2 μM. A non-targeting siRNA (NT, GE Dharmacon, D-001810-10) and an siRNA targeting MxA (MX1 gene) (Qiagen, SI0559538) were used as negative and positive controls respectively.

### Protein precipitation

A549-Strep-MxA and A549-Strep-GFP cells were seeded in 15 cm dishes and treated with doxycycline (1 μg/mL) for 24 h before infection with the H5N1 Renilla luciferase virus (MOI of 8). 24 h later, the cells were washed with PBS and lysed in 1 mL cold lysis buffer [50 mM Tris-HCl, pH7.4, 150 mM NaCl, 1 mM EDTA, 0.5% NP-40, 1× protease inhibitor cocktail (EDTA-free, Roche), 1× phosphatase inhibitor cocktail (PhosStop, Roche)]. To assist with lysis of the nuclear compartment, the lysates were frozen at −80 °C and thawed prior to immunoprecipitation.

Lysates were clarified by centrifugation at 3500 × g for 20 min. 50 µL of cleared supernatant was retained as an ‘Input’ sample. The remaining 950 µL was added to 550 µL IP buffer (50 mM Tris-HCl, pH7.4, 150 mM NaCl, and 1 mM EDTA) containing 20 µL equivalent bead volume of Strep-Tactin Sepharose beads (IBA Lifesciences). Affinity tag binding proceeded with rotation at 4 °C for 2 hours. Beads were centrifuged at 300 × g for 3 minutes. 50 µL of cleared supernatant was retained as an ‘Unbound Flow Through’ sample, and the remaining supernatant was discarded. The beads were washed twice in IP buffer containing 0.05% NP-40 and twice in IP buffer with no detergent.

Streptactin-purified proteins were reduced and alkylated on beads with 20 µL reduction-alkylation buffer [50 mM Tris-HCl, pH8.0, 2 M Urea, 1 mM DTT, 3 mM iodoacetamide] and incubated in the dark for 45 minutes with gentle shaking. An additional 3 mM DTT was added to quench the reaction, and proteins were digested with 0.75 µg trypsin (Invitrogen) overnight at 37 °C. The next day, the beads were centrifuged at 300 × g for 3 minutes. The peptide-containing supernatant was collected and formic acid was added to a final concentration of 1% to acidify the peptides. Peptides were desalted using Agilent OMIX C18 10 µL tips according to the manufacturer’s protocol with the following modifications. Briefly, tips were conditioned with 50% acetonitrile, 0.1% formic acid and then equilibrated by two rinses with 0.1% formic acid. Peptides were bound by repeated pipetting, rinsed twice in 0.1% formic acid, and eluted in 50% acetonitrile. A second elution in 90% acetonitrile was used to ensure complete recovery. Peptides were dried under vacuum centrifugation and suspended in 12 µL of 3.0% acetonitrile, 0.1% formic acid.

### Protein identification by liquid chromatography tandem mass spectrometry (LC MS/MS)

Digested peptides were subjected to LC-MS/MS analysis using an Easy-nLC 1000 coupled to a dual-pressure linear ion trap (Velos Pro) Orbitrap Elite mass spectrometer (Thermo Fisher Scientific, San Jose, CA). Online LC separation was performed using a 75 µm × 25 cm fused silica IntegraFrit capillary packed with 1.9 µm Reprosil-Pur C18 AQ reversed-phase resin (Dr. Maisch-GmbH). Peptides were eluted by a gradient of 5% to 30% acetonitrile in 0.1% formic acid in 110 minutes delivered at a flow rate of 300 nL/minute. For each cycle, one full MS scan (150–1500 m/z, resolution of 120,000) in the Orbitrap was followed by 20 data-dependent MS/MS scans fragmented by normalized collision energy (setting of 35%) and acquired in the linear ion trap. Target ions already acquired in MS/MS scans were dynamically excluded for 20 seconds. Raw MS files were analyzed by MaxQuant^31^ version 1.3.0.3 and MS/MS spectra searched by the Andromeda search engine^83^ against a database containing SwissProt human and influenza protein sequences (20,226 total)^84^. All runs for a given bait were analyzed simultaneously to maximize the “match between runs” algorithm available on MaxQuant. Multiplicity was set to 1 and a false discovery rate of 0.01 imposed for peptide and protein identification. Normalization of raw peptide intensities and protein level abundance inference were calculated using the linear mixed-effects model built into the MSstats R package version 3.3.10^30^. Proteins that appeared in a single biological replicate were excluded from further analysis.

### Network analysis

The results from both genomic and proteomic screens were combined and analyzed using Ingenuity Pathway Analysis (IPA®, Qiagen) to look at the gene ontology, canonical pathways, regulators and protein-protein interaction enrichment. Considering all terms with p-value < 0.01, we identified 13 factors present in all enrichment categories. A protein-protein interaction network showing the physical interactions within these 13 factors was also generated using IPA® (Qiagen).

### siRNA validation

A549-MxA cells were seeded in 96-well plates (7600 cells/well) and reverse transfected with siRNA using Lipofectamine RNAiMAX (0.2 μL/well) at a final siRNA concentration of 30 nM. 72 hours after transfection cells were infected with the H5N1 Renilla luciferase reporter virus at an MOI of 8 for 24 hours. Luciferase activity was analyzed by adding 20 µl luciferase substrate (Renilla-Glo Luciferase Assay System, Promega) per well and read using the Glomax multiplus plate reader (Promega) or the Infinite M200 Microplate reader (Tecan). All experiments were performed at least in technical triplicates.

### Transcriptome analysis (RNA-Seq)

2 × 10^5^ A549-MxA cells were transfected with a non-targeting control siRNA or a SMARCA2-targeting siRNA (Dharmacon D-017253-01) in triplicate. 72 h after transfection cells were infected with H5N1 wild type virus (A/Thailand/1(KAN-1)/2004) at an MOI of 1 for 24 h. Cells were harvested in DNA/RNA Shield (Zymo Research, cat. #R1100-50) and total RNA was extracted using Quick-RNA Miniprep Plus Kit from Zymo Research (cat. #R1057). 3 µg total RNA from each sample was treated with the Ribo-Zero™ Magnetic Gold Kit (Human/Mouse/Rat) from Illumina (cat. #MRZG126) and libraries were prepared from rRNA depleted samples using ScriptSeq™ v2 RNA-Seq Library Preparation Kit from Illumina (Cat. #SSV21106). RNA-Seq libraries were sequenced on an Illumina HiSeq to a sequencing depth of 40–50 million reads (50 bp single-read).

### Bioinformatics analysis of RNA-Seq

Illumina HiSeq. 50 bp single-end reads from RNA-Seq libraries were first adaptor trimmed and then analyzed using the TopHat and Cufflinks software. TopHat (v2.0.13) was utilized for alignment of short reads to the reference genome. To analyze gene expression, Cufflinks (v2.2.1) was utilized for transcript assembly and differential expression, and cummeRbund (v2.0.0) for visualization of differential analysis. Default parameters were used.

Additional methods can be found in the supplementary information.

### Data availability

Any reagent will be shared and distributed to other investigators upon request for research purposes and upon signing of a standard material transfer agreement from the relevant institute if necessary.

## Electronic supplementary material

Supplementary Information

Dataset 1

Dataset 2

Dataset 3

Dataset 4

Dataset 5
