# Author Correction: SMARCA2-regulated host cell factors are required for MxA restriction of influenza A viruses

**DOI:** 10.1038/s41598-018-25858-y

**Published:** 2018-05-14

**Authors:** Dominik Dornfeld, Alexandra H. Dudek, Thibaut Vausselin, Sira C. Günther, Judd F. Hultquist, Sebastian Giese, Daria Khokhlova-Cubberley, Yap C. Chew, Lars Pache, Nevan J. Krogan, Adolfo Garcia-Sastre, Martin Schwemmle, Megan L. Shaw

**Affiliations:** 1 Institute of Virology, Medical Center, University of Freiburg, 79104 Freiburg, Germany; 2 Spemann Graduate School of Biology and Medicine, University of Freiburg, 79104 Freiburg, Germany; 3 Faculty of Medicine, University of Freiburg, 79104 Freiburg, Germany; 4 Faculty of Biology, University of Freiburg, 79104 Freiburg, Germany; 5 Department of Microbiology, Icahn School of Medicine at Mount Sinai, New York, NY 10029 USA; 6 Global Health and Emerging Pathogens Institute, Icahn School of Medicine at Mount Sinai, New York, NY 10029 USA; 7 Department of Medicine, Division of Infectious Diseases, Icahn School of Medicine at Mount Sinai, New York, NY 10029 USA; 8 Zymo Research Corp, Irvine, CA 92614 USA; 9 Quantitative Biosciences Institute, QBI, Department of Cellular and Molecular Pharmacology, University of California, San Francisco, San Francisco, CA 94158 USA; 10 J. David Gladstone Institutes, San Francisco, CA 94158 USA; 11 Sanford Burnham Prebys Medical Discovery Institute, Infectious and Inflammatory Disease Center, 10901 North Torrey Pines Road, La Jolla, CA 92037 USA

Correction to: *Scientific Reports* 10.1038/s41598-018-20458-2, published online 01 February 2018

Lars Pache was omitted from the author list in the original version of this Article. This has been corrected in the PDF and HTML versions of the Article and in the accompanying Supplementary Information.

The Author Contributions section now reads:

“D.D., M.S. and M.L.S. conceived and designed the experiments. D.D., A.H.D., T.V, S.C.G., J.F.H., S.G., D.K.-C, Y.C.C and L.P. performed the experiments. D.D., M.S., M.L.S., A.H.D., T.V., J.F.H., D.K.-C., Y.C.C., L.P., N.J.K and A.G.-S. analyzed the data. D.D., M.L.S., M.S. and A.G.-S wrote the paper.”

