# Supplementary material for: SMARCA2-regulated host cell factors are required for MxA restriction of influenza A viruses
Source: Sci Rep. 2018 Feb 1;8:2092. doi: 10.1038/s41598-018-20458-2 (PMC5794779; doi:10.1038/s41598-018-20458-2)
Supplement: Supplementary file 1 — Supplementary Information [file 41598_2018_20458_MOESM1_ESM.pdf]

SMARCA2-regulated host cell factors are required for MxA restriction of influenza A viruses

Dominik Dornfeld[a,c,1], Alexandra H. Dudek[a,b,c,d,1], Thibaut Vausselin[e,1], Sira C. Günther[a,c], Judd F. Hultquist[i,j], Sebastian Giese[a,c], Daria Khokhlova-Cubberley[h], Yap C. Chew[h], Lars Pache[k], Nevan J. Krogan[i,j], Adolfo Garcia-Sastre[e,f,g,*], Martin Schwemmle[a,c,*], Megan L. Shaw[e,*]

[a]Institute of Virology, Medical Center, [b]Spemann Graduate School of Biology and Medicine, [c]Faculty of Medicine, [d]Faculty of Biology, University of Freiburg, 79104 Freiburg, Germany, [e]Department of Microbiology, [f]Global Health and Emerging Pathogens Institute,[g]Department of Medicine, Division of Infectious Diseases, Icahn School of Medicine at Mount Sinai, New York, NY 10029, USA, [h]Zymo Research Corp, Irvine, CA 92614, USA, [i]Quantitative Biosciences Institute, QBI, Department of Cellular and Molecular Pharmacology, University of California, San Francisco, San Francisco, CA 94158, USA, [j]J. David Gladstone Institutes, San Francisco, CA 94158, USA, [k]Sanford Burnham Prebys Medical Discovery Institute, Infectious and Inflammatory Disease Center, La Jolla, CA 92037, USA.

*[1]contributed equally, *corresponding authors*

SUPPLEMENTARY INFORMATION

SUPPLEMENTARY MATERIALS AND METHODS

Media. Opti-Minimum Essential Medium (OptiMEM®, Gibco Life TechnologiesTM) and Dulbecco Modified Eagle Medium (DMEM) with and without phenol red (Corning Cellgro) were used for cell culture.

Reagents. DharmaFECT4 (GE Dharmacon) and Lipofectamine® RNAiMAX (Invitrogen) were used for the siRNA transfection at a ratio of 1:4 and 1:6 µL per pmol of siRNA respectively. Doxycycline (Sigma, D9891) was used at 1 µg/mL. Human chimeric IFN-αB/D was used at either 1000 U/mL or 25 U/mL (78).

Antibodies. Anti-JAK1 antibody (BD Bioscience, 610232) was used at a 1:350 dilution. Anti-MxA antibody (79), anti-SMARCA2 (Abcam, ab15597) and anti-actin (Sigma-Aldrich A3853) antibodies were used at a 1:1000 dilution. Anti-NP (GTX,125989) was used at a 1:2000 dilution. Goat anti-rabbit-HRP and rabbit anti-mouse-HRP (Jackson Immunoresearch) were used at a dilution of 1:5000.

A549-Strep-MxA cell line generation. The coding sequence for MxA (NM_001144925) was cloned with a N-terminal 2xStrep tag into pLVX-TetOne-Puro (Clontech) and a 2xStrep-eGFP construct (coding sequence from GQ404376.1) was cloned analogously. Lentiviruses were generated by co-transfection of HEK293T cells with each of the pLVX-TetOne-Puro lentiviral plasmids described above, a Gag-Pol packaging construct, and plasmid encoding the VSV-G envelope (pMD2.G, Addgene). The supernatant was collected and lentiviruses were precipitated with 8.5 % PEG-6000 and 0.3M NaCl at

4°C. Viruses were pelleted at 3500 rpm for 20 minutes, suspended in 1 mL 1xPBS, and aliquoted for storage at -80°C. To generate stable cells, A549 cells were transduced with the lentiviral concentrate, and after 48 hours, the media was replaced and supplemented with 1 µg/mL puromycin. Cells were allowed to expand for 72 hours to select for successfully transduced cells.

Immunofluorescence analysis. A549 cells were seeded on coverslips in a 24 well plate transfected with siRNA as described above. 96 h post siRNA transfection cells were washed with PBS containing calcium and magnesium chloride and fixed with paraformaldehyde (4%) for 15 min at RT. Fixed cells were washed three times with PBS, permeabilized with Triton-X-100 (0.5%) for 5 min at RT and washed again three times with PBS. The staining was performed with primary antibodies (mouse anti-MxA, 1:1000 ; rabbit anti-NP, 1:2000, GeneTex) diluted in 5 % Normal Goat Serum (NGS) for 1.5 h at RT. Coverslips were washed four times with PBS and incubated with the secondary antibodies (CyTM3-conjugated goat anti-mouse, 1:500, Jackson ImmunoResearch; CyTM2-conjugated goat anti-rabbit, 1:500, Jackson ImmunoResearch) diluted in 5 % NGS for 30 min at RT in the dark. Cells were washed once with PBS, stained with DAPI at a concentration of 1:10000, followed by two further wash steps with PBS. Coverslips were finally dipped in Milli-Q water, mounted on glass slides using Immuno-Mount (Thermo Scientific) and incubated overnight at RT. Images were taken with the Axio Observer.Z1 (Zeiss) and analyzed using AxioVision software.

Flow cytometry analysis. Intracellular staining of A549 cells was performed using the FoxP3/Transcription Factor Staining Buffer Set (eBioscience). Cells were collected from

a 24-well plate and centrifuged at 900 rpm for 5 min. The cell pellet was resuspended in 500 µl of 1 x Fixation/Permeabilization solution and incubated for 30 min at 4 °C. Cells were then washed twice with 1 x permeabilization buffer, centrifuged at 1600 rpm for 10 min and incubated with the primary antibodies (mouse anti-MxA, 1:100 (79); rabbit anti-NP, 1:100, GeneTex) in 50 µl of 1 x permeabilization buffer for 30 min at 4 °C in the dark. After washing the cells twice, they were incubated with the secondary antibodies (Brilliant Violet 421TM-conjugated donkey anti-rabbit, IgG, 1:50, BioLegend; PE conjugated rat anti-mouse, IgG1, κ, 1:50, BD Biosciences) for 30 min at 4 °C in the dark. Washed cells were then resuspended in 100 µl of 2% PFA and analyzed using the FACSCantoTM (BD Bioscience). The results were analyzed using FlowJo software.

Cell viability. Cellular cytotoxicity was assessed using the 3-(4,5-Dimethylthiazol-2-yl)-2,5- diphenyltetrazolium bromide (MTT) assay (Roche) following manufacturer's instructions. Absorbance was measured at 565 nm using the Tecan plate reader.

Statistical analysis. Results from the primary siRNA screen were analyzed based on the Z-score calculation. Results from the secondary siRNA screen were analyzed using the redundant siRNA activity method (RSA) as previously described (27). For the siRNA screens the assay performance of each plate was assessed by calculating the strictly standardized mean difference (SSMD) (26) and validated if the SSMD was above 3. RNA-Seq data was analyzed as described above. For other experiments Student's t-test analysis was performed.

Data Availability. Any reagent will be shared and distributed to other investigators upon request for research purposes and upon signing of a standard material transfer agreement from the relevant institute if necessary.

SUPPLEMENTARY FIGURES

Supplementary Figure S1

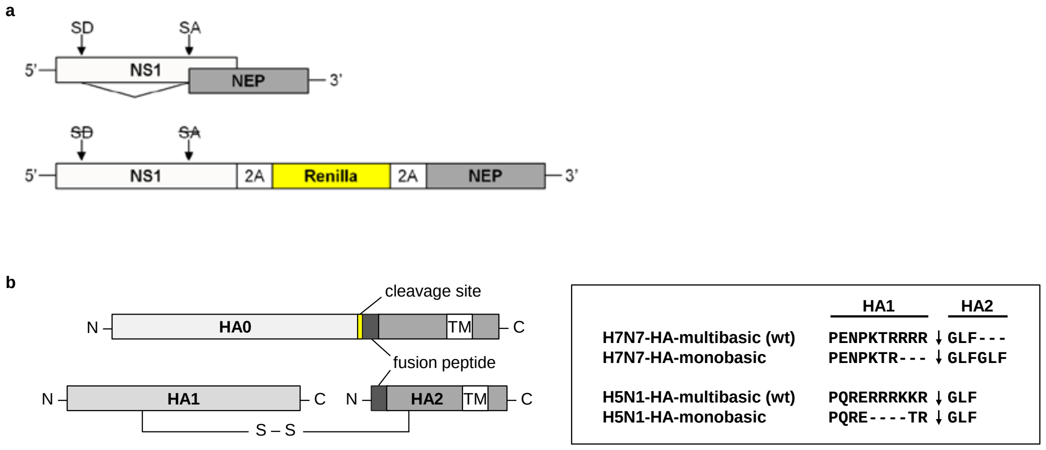

Supplementary Figure S1. Construction of Renilla-expressing reporter viruses with monobasic HA cleavage site. (**a**) The wild type NS genomic segment (upper ribbons) encodes NS1 and NEP from two overlapping ORFs. Expression of NEP is mediated by splicing. By separating NS1 and NEP, silencing the splice donor (SD) and splice acceptor (SA) site, a Renilla-encoding sequence (RL) can be introduced between the NS1 and NEP ORFs. NS1 and the RL are co-translationally cleaved by the 2A peptide of porcine teschovirus-1 (2A). Separation of RL from NEP is achieved by a second 2A peptide (2A*), whose coding sequence was codon optimized to prevent homologous recombination. (**b**) Schematic illustration of the HA0 protein and the cleaved form comprising disulfide-linked HA1 (light grey) and HA2 (dark grey) with the fusion peptide accessible. The cleavage site is highlighted in yellow. Right panel: Alignment of amino acid sequences of the HA cleavage site from the influenza A virus strain A/seal/Massachusetts/1/1980 (H7N7) and A/Vietnam/1203/2004 (H5N1). The arrow indicates the cleavage site linking HA1 and HA2. In case of the wild type HA, a multibasic cleavage site composed of a stretch of lysine (K) and/or arginine (R) residues is present at the cleavage site. By deletion of the basic amino acids, a monobasic HA cleavage site was generated. TM = transmembrane domain.

Supplementary Figure S2

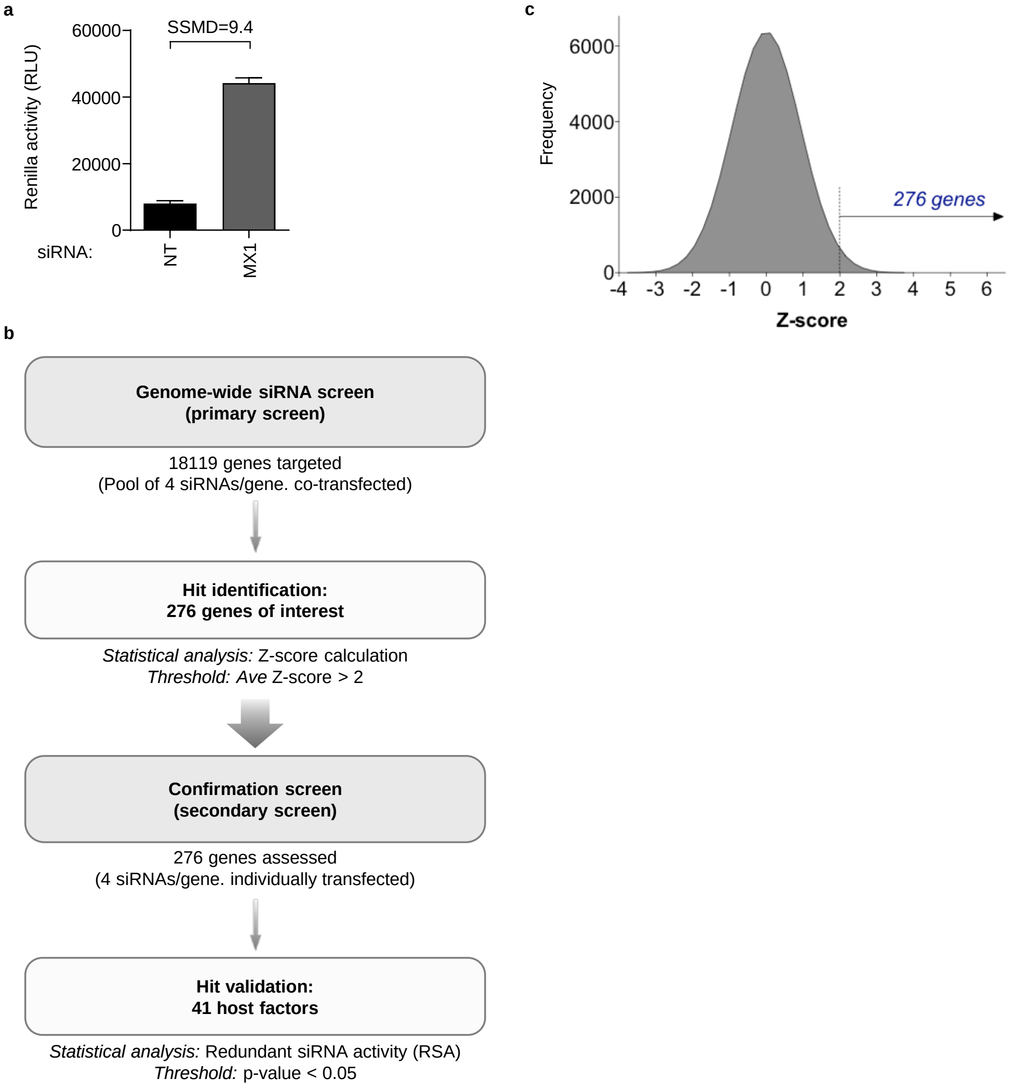

Supplementary Figure S2. RNAi screen. (**a**) Quality control prior to genome-wide siRNA screen to assure sufficient assaying window. The strictly standardized mean difference (SSMD) was calculated using the negative (NT) and positive (MX1) controls. (**b**) Workflow illustration of the primary and secondary siRNA screens. In the primary genome-wide screen, siRNA pools (4 siRNAs per gene) targeting 18119 genes were assessed for effects on virus replication in A549-MxA cells. 276 hits were identified based on an average Z-score > 2 across triplicates. A secondary screen was performed on these genes where the 4 siRNAs from the pool were individually assessed. 41 hits were identified with $p < 0.05$. (**c**) Distribution of the 18119 genes based on their Z-score. RLU = Relative Light Units.

Supplementary Figure S3

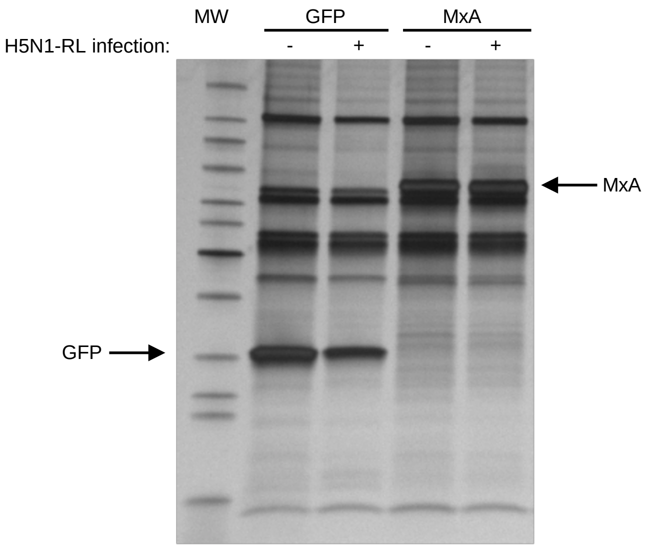

Supplementary Figure S3. Proteomic screen. A549-Strep-GFP and A549-Strep-MxA cells were treated with doxycycline to induce Strep-GFP or Strep-MxA, respectively, and infected with H5N1-RL at an MOI of 1 for 24 h. Strep-tagged proteins were precipitated with streptavidin and the eluates visualized by SDS-PAGE and silver stain. Positions of GFP and MxA are indicated. MW = Molecular Weight

Supplementary Figure S4

a

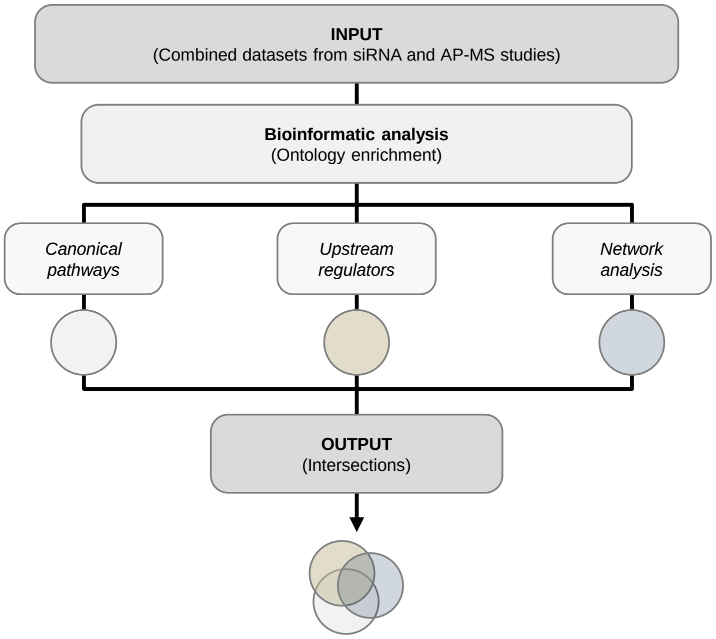

b

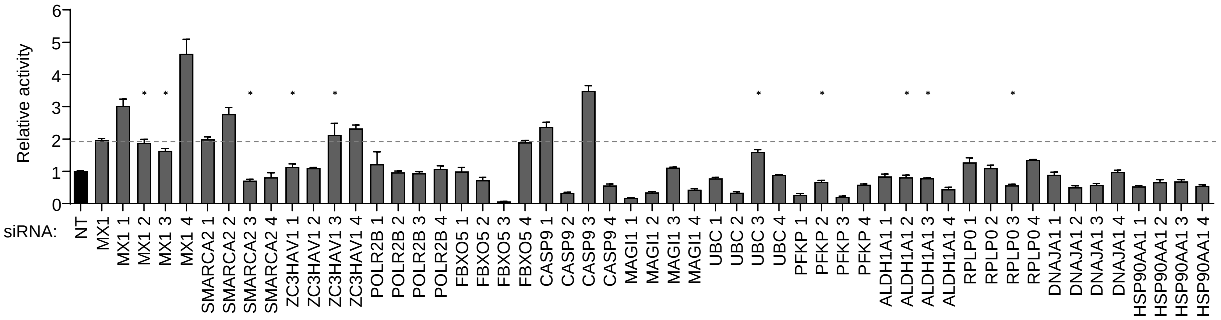

Supplementary Figure S4. Bioinformatic analysis and siRNA validation. (**a**) Workflow illustration of the bioinformatic analysis. Results from both RNAi and proteomic screens were combined and analyzed to look at the canonical pathways, upstream regulators and protein-protein interaction enrichment. For each analysis, a list of enriched terms was obtained. We considered all terms with p-value < 0.01 and their corresponding genes. By overlaying them we identified 13 factors shared in all enrichment lists. (**b**) The 13 factors were silenced individually on A549-MxA cells to evaluate the effect on reporter virus activity. For this purpose A549-MxA cells were transfected with either a NT control siRNA, an MxA-targeting (MX1) siRNA (targeting the MX1 gene and using the siRNA originally utilized for the genome-wide siRNA screen (Qiagen SI05459538) or one of 4 siRNAs targeting the respective gene identified by the bioinformatic analysis. 72 h post transfection A549-MxA cells were infected with H5N1-RL at an MOI of 8. 24 h post infection reporter activity was evaluated using Renilla-Glo substrate (Promega). All data were normalized to the NT control. Factors whose

depletion led to a stronger increase of viral replication than MX1 depletion (> 90%; dashed line) for at least 2 out of 4 siRNAs were considered validated (3 technical replicates). Student's t-test was performed to determine the *P* value. **P* < 0.05, ***P* < 0.01, ****P* < 0.001. AP-MS = Affinity Purification-Mass Spectrometry.

Supplementary Figure S5

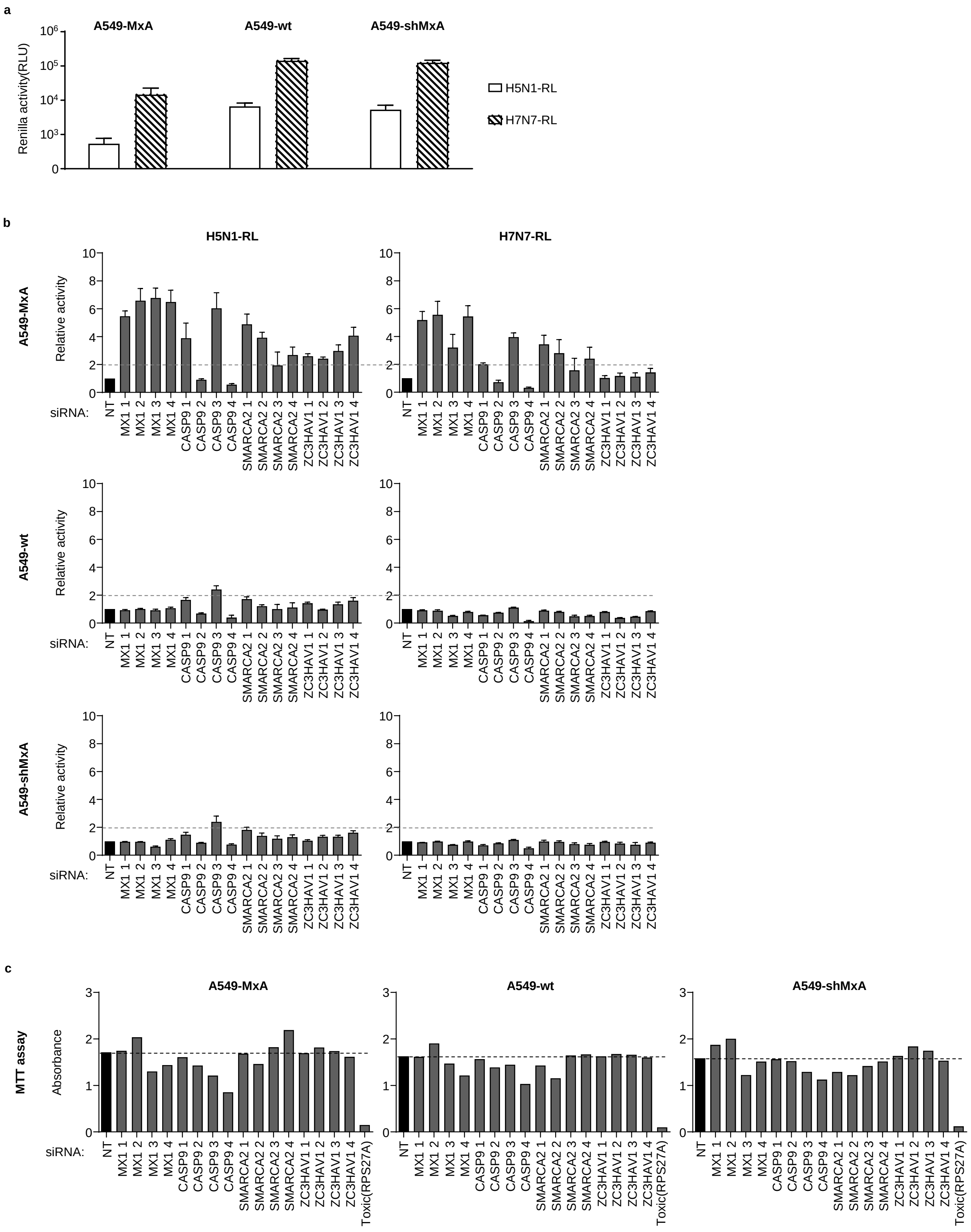

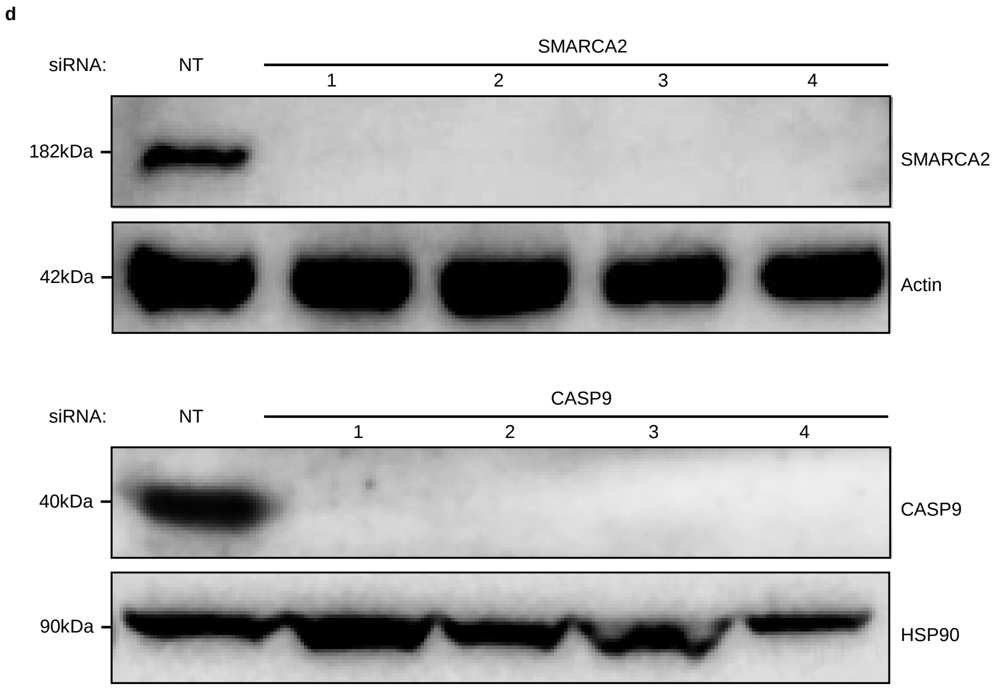

Figure S5: SMARCA2 and CASP9 decrease viral replication in an MxA-dependent manner. (**a**) A549-MxA, A549 wild type cells (A549-wt) or A549 cells stably expressing a short hairpin RNA targeting MxA (A549-shMxA) were seeded as for siRNA transfections. After 72 h, using an MOI of 0.3, cells were infected with H5N1-RL or an H7N7 Renilla-expressing virus (H7N7-RL). After 24 h virus reporter activity was measured using Renilla-Glo (Promega) substrate. The data of 3 independent experiments is presented (6 technical replicates per experiment). (**b**) To test MxA dependence A549-MxA, A549-wt or A549-shMxA cells were transfected with a NT control siRNA or with one of four siRNAs targeting either MxA (MX1), CASP9, SMARCA2 or ZC3HAV1. 72 h post transfection, using an MOI of 0.3, cells were infected with either H5N1-RL or H7N7-RL. After 24 h virus reporter activity was measured using Renilla-Glo (Promega) substrate. All data were normalized to the NT control siRNA. The data of 3 independent experiments is presented (4 technical replicates). The dashed line indicates the threshold of 2-fold increase over the NT control. (**c**) Cell viability 72 h post siRNA transfection as determined by MTT assay. A toxic siRNA targeting ribosomal protein RPS27A (Toxic(RPS27A)) was used as control. A dashed line indicates the absorbance after non-targeting control siRNA (NT) transfection. (**d**) Knockdown efficiency of the 4 individual siRNAs targeting SMARCA2 or CASP9, respectively, was determined after 72 h. Cell lysates were subjected to western blot analysis. Full-length blots are presented in Supplementary Figure S8d and S8e. RLU = Relative Light Units.

Supplementary Figure S6

a

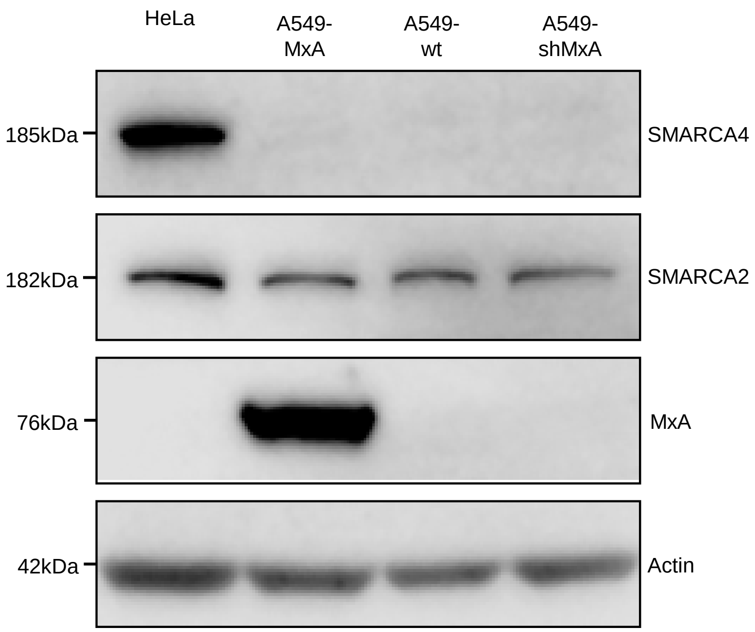

b

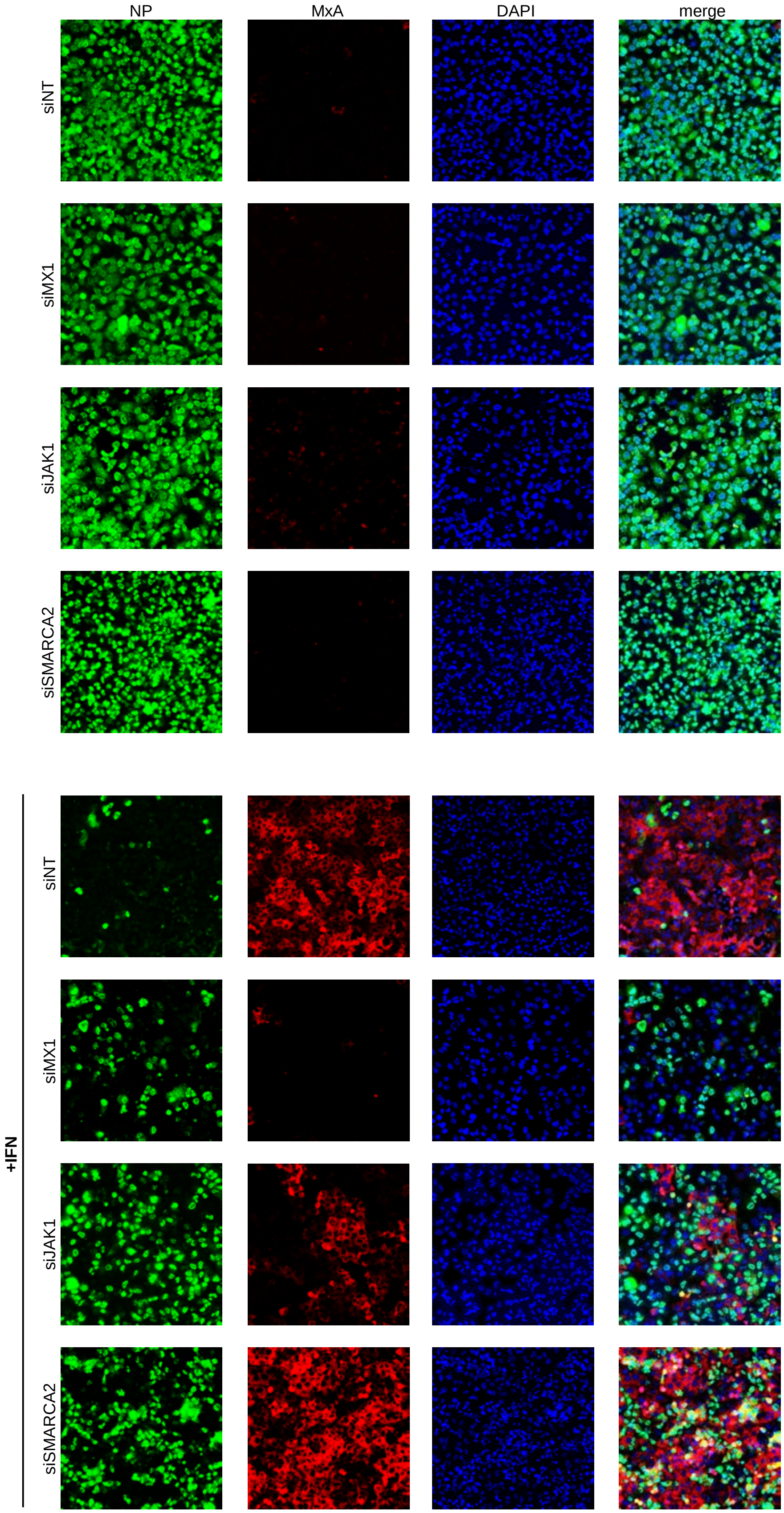
NP
MxA
DAPI
merge
siNT
siMX1
siJAK1
siSMARCA2
+IFN
siNT
siMX1
siJAK1
siSMARCA2

Figure S6: Absence of SMARCA4 in A549 cells and increased IAV susceptibility of A549 cells expressing MxA. (**a**) HeLa, A549-MxA, A549-wt and A549-shMxA cells were lysed and subjected to western blot analysis. Detection of Actin was used to control loading. Full-length blots are presented in Supplementary Figure S8f. (**b**) A549-wt cells were transfected with a non-targeting (siNT) siRNA, or siRNAs targeting MxA (siMX1), JAK1 (siJAK1), or SMARCA2 (siSMARCA2) (GE Dharmacon D-017253-01). 48 h post transfection the cells were either treated, or not, with IFN-α (1000 U/mL) and 24 h later were infected with H7N7-RL at an MOI of 1 for 24 h. For immunofluorescence analysis MxA (red) and NP (green) were stained with specific antibodies. DAPI was used to counterstain the nucleus (blue).

Supplementary Figure S7

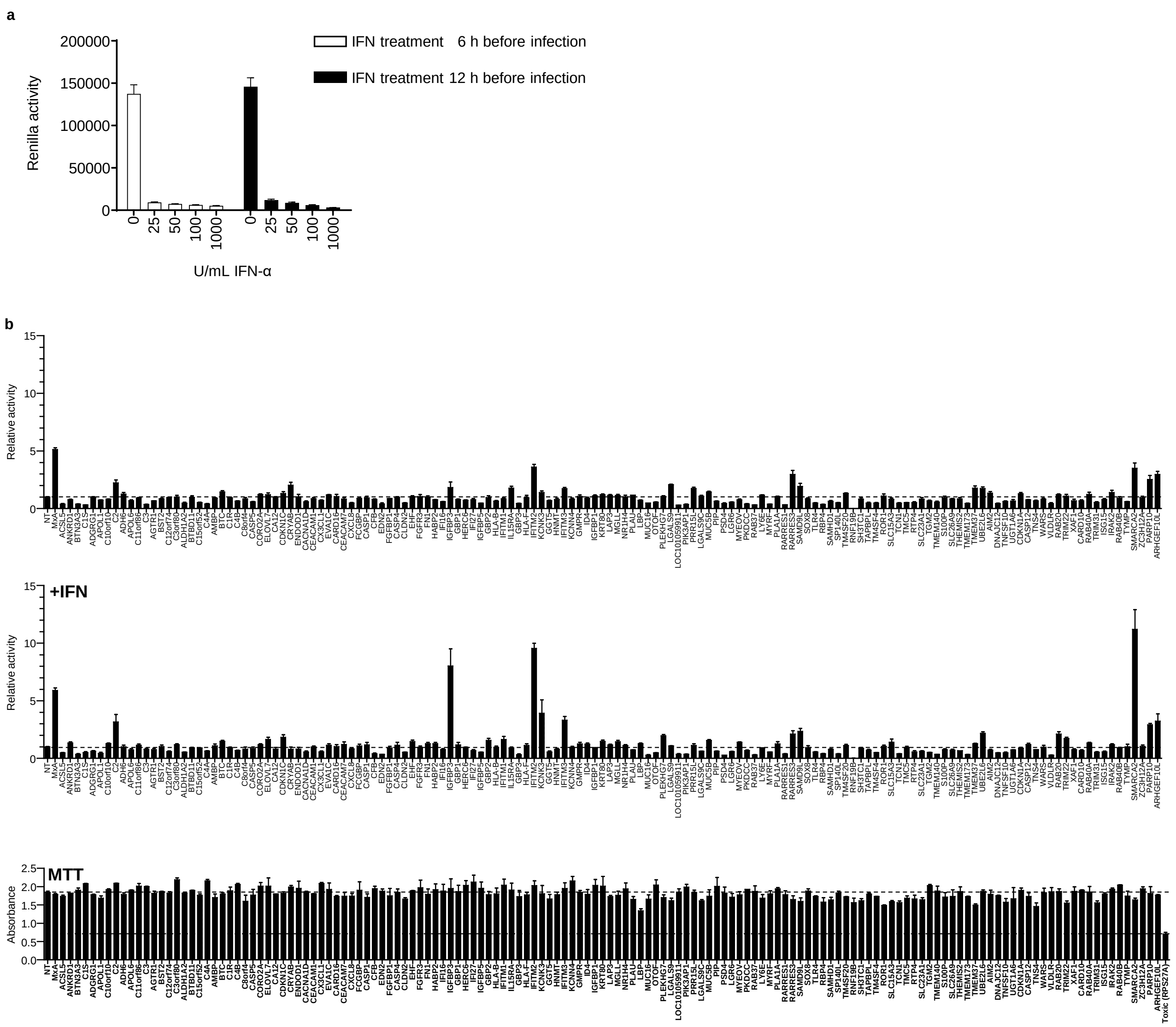

Figure S7: Identification of SMARCA2-regulated MxA cofactors. (**a**) A549-MxA cells were seeded as for knockdown experiments. 6 h or 12 h before infection cells were treated with different concentrations of IFN-α. 72 h post seeding cells were infected with H7N7-RL. 24 h later read-out was performed using Renilla-Glo substrate (Promega) (12 technical replicates). (**b**) Host cell factors whose mRNA abundance was strongly decreased after SMARCA2 knockdown were silenced in A549-MxA cells using siRNA pools. 66 h after siRNA transfection A549-MxA cells were either treated, or not, with IFN-α (25 U/mL) and infected 6 h later with the H7N7-RL reporter virus (MOI=0.3). After 24 h virus reporter activity was measured using Renilla-Glo (Promega) substrate. All data was normalized to the NT control siRNA (3 technical replicates). Student’s t-test

was performed to determine the P value *$P < 0.05$, **$P < 0.01$, ***$P < 0.001$. Cell viability 72 h post siRNA transfection was determined by MTT assay. A toxic siRNA targeting RPS27A (Toxic) was used as a control.

Supplementary Figure S8

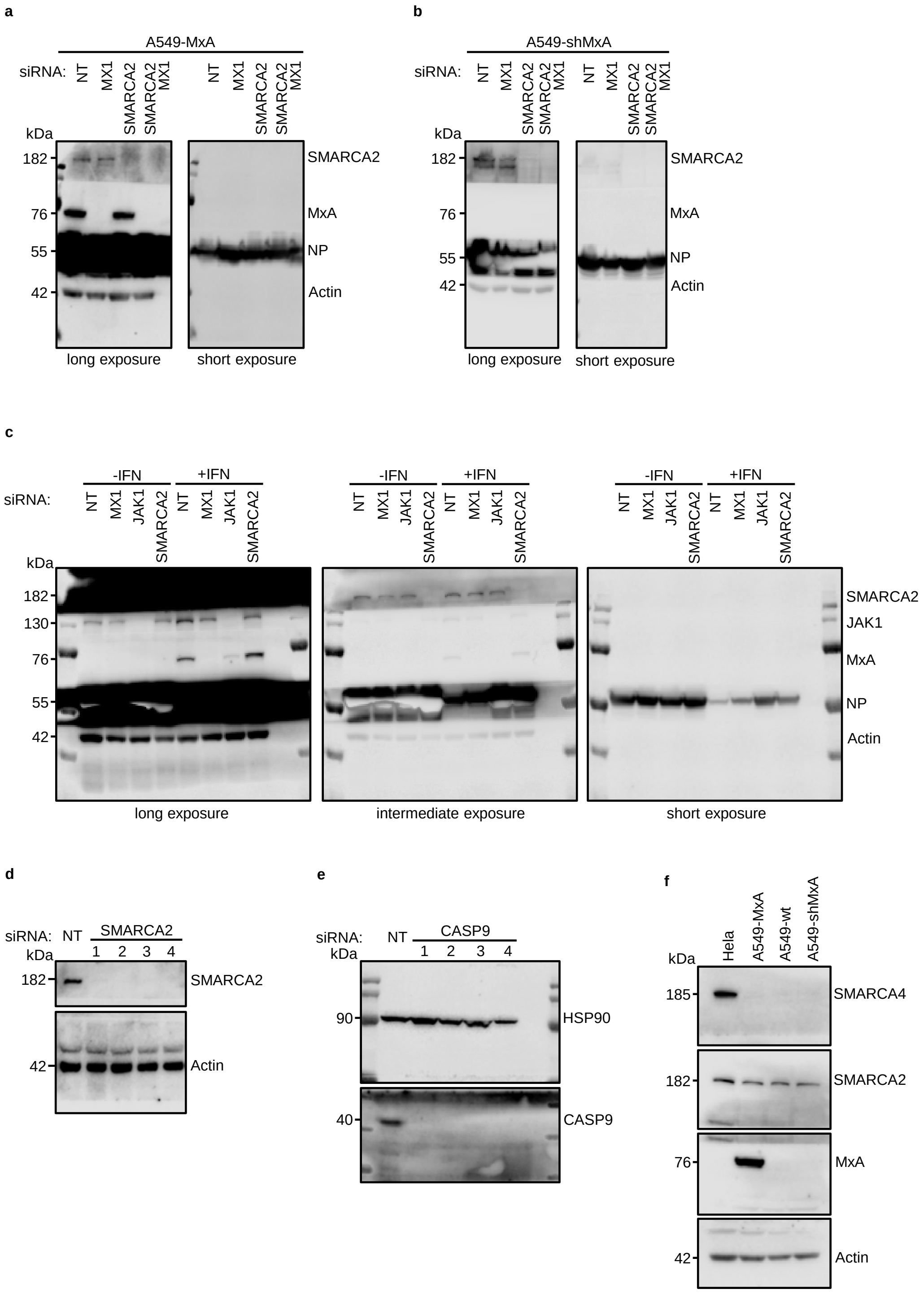

Supplementary Figure S8: Uncropped scans. (a) Uncropped scans of the left panel (A549-MxA) of Fig. 1f. The membrane was cut horizontally and stained against SMARCA2, MxA, NP or Actin. To optimally visualize all detected proteins, the exposure

time was varied (long exposure / short exposure). (b) Uncropped scans of the right panel (A549-shMxA) of Fig. 1f. Cutting, staining and scanning was performed as described in (a). (c) Uncropped scans of Fig. 2a. The membrane was cut horizontally and stained against SMARCA2, JAK1, MxA, NP or Actin. To optimally visualize all detected proteins, the exposure time was varied (long exposure / intermediate exposure / short exposure). (d) Uncropped scans of Supplementary Figure S5d (upper panel). The membrane was cut horizontally and stained against SMARCA2 or Actin. (e) Uncropped scans of Supplementary Figure S5d (lower panel). The membrane was cut horizontally and stained against HSP90 or CASP9. (f) Uncropped scans of Supplementary Figure S6. The membrane was cut horizontally and stained against SMARCA4, SMARCA2, MxA and Actin.
